# Supplementary material for: Genetic dissection of the glutamatergic neuron system in cerebral cortex
Source: Nature. 2021 Oct 6;598(7879):182–7. doi: 10.1038/s41586-021-03955-9 (PMC8494647; doi:10.1038/s41586-021-03955-9)
Supplement: Supplementary file 1 — This file contains a list of abbreviations, legends for the Supplementary Tables and Videos, and instructions for Data Portal use. [file 41586_2021_3955_MOESM1_ESM.pdf]

nature portfolio

https://doi.org/10.1038/s41586-021-03955-9

Supplementary information

Genetic dissection of the glutamatergic neuron system in cerebral cortex

In the format provided by the authors and unedited

nature portfolio

https://doi.org/10.1038/s41586-021-03955-9

Supplementary information

Genetic dissection of the glutamatergic neuron system in cerebral cortex

In the format provided by the authors and unedited

SUPPLEMENTARY INFORMATION

Genetic dissection of the glutamatergic neuron system in cerebral cortex

Katherine S. Matho[1], Dhananjay Huilgol[1,2#], William Galbavy[1,3#], Miao He[1#,a], Gukhan Kim[1#], Xu An[1,2], Jiangteng Lu[1,b], Priscilla Wu[1], Daniela J. Di Bella[4], Ashwin S. Shetty[4], Ramesh Palaniswamy[1], Joshua Hatfield[1,2], Ricardo Raudales[1,3], Arun Narasimhan[1], Eric Gamache[1], Jesse Levine[1,6], Jason Tucciarone[1,6,c], Eric Szelenyi[1], Julie A. Harris[6], Partha P. Mitra[1], Pavel Osten[1], Paola Arlotta[4,5], Z. Josh Huang[1,2*]

[1]Cold Spring Harbor Laboratory, Cold Spring Harbor, New York 11724, USA.

[2]Department of Neurobiology, Duke University Medical Center, Durham, NC

[3]Program in Neuroscience, Department of Neurobiology and Behavior, Stony Brook University, Stony Brook, NY, 11794, USA.

[4]Department of Stem Cell and Regenerative Biology, Harvard University, Cambridge, MA 02138, USA.

[5]Stanley Center for Psychiatric Research, Broad Institute of MIT and Harvard, Cambridge, MA 02138, USA.

[6]Program in Neuroscience and Medical Scientist Training Program, Stony Brook University, New York 11790, USA.

[7]Allen Institute for Brain Science, Seattle, WA, 98109, USA.

[*] corresponding author: josh.huang@duke.edu
[#] These authors contributed equally to this work.
Current address:
[a]Institutes of Brain Science, State Key Laboratory of Medical Neurobiology and MOE Frontiers Center for Brain Science, Fudan University, Shanghai 200032, China.
[b] Shanghai Jiaotong University Medical School, Shanghai, China.
[c] Department of Psychiatry, Stanford University School of Medicine, Palo Alto, CA, US

TABLE OF CONTENTS

**Page 3**	**List of Abbreviations**
**Page 6**	**List of Supplementary Tables**
**Page 7**	**List of Supplementary Videos**
**Page 8**	**Instructions for Data Portal Use**

LIST OF ABBREVIATIONS

AAV, adeno-associated virus
ACA, anterior cingulate area
AI, Agranular Insula
AMY, amygdala
AP, antero-posterior coordinate relative to Bregma
APT, anterior pretectal nucleus
AUDp, Primary auditory cortex
BICCN, Brain Initiative Cell Census Network
BIL, BICCN Brain Image Library
BLA, basolateral amygdala
BS, brainstem
Cb, cerebellum
cc, corpus callosum
c-ECT- contralateral ectorhinal cortex
c-MO- contralateral motor cortex
CNU, cerebral nuclei
cp, cerebral peduncle
CPN, callosal projection IT PyN
cSSp, contralateral primary somatosensory cortex
cSp5, contralateral spinal trigeminal nucleus
CST, corticospinal tract
CT, corticothalamic
CTB, Cholera toxin subunit B
CTX, cerebral cortex
ET, extratelencephalic
HAR, human accelerated region
HP, hippocampus
HSV, herpes simplex virus
HT, hypothalamus
HPF, hippocampal formation
i-ECT, ipsilateral ectorhinal cortex
ILA- infralimbic area
i-MO, ipsilateral motor cortex
iMOs, ipsilateral secondary motor cortex
IP, intermediate progenitor
IS, intersection/subtraction
IT, intratelencephalic
KI, knock-in

LA, lateral amygdala
MD, mediodorsal nucleus of the thalamus
MO, motor cortex
MOp, Primary motor cortex
MOs, secondary motor cortex
nRG, neurogenic radial glia
OB, olfactory bulb
OLF, olfactory areas
ORBm, Orbital area medial part
ORBl/vl, Orbital area lateral/ventral lateral parts
PAL, pallidum
PAG, periaqueductal gray
PL, prelimbic area
Pn, pons
PO/POm, posteromedial complex of the thalamus
PT, pyramidal tract
PV, parvalbumin
PyN, pyramidal neuron
retroAAV, retrograde adeno-associated virus
RG, radial glial progenitor
RT, reticular nucleus of the thalamus
SC, superior colliculus
SCPN, subcerebral projection neurons
Sp5, spinal trigeminal nucleus
Spd/Sp. cord, spinal cord
SSp, Primary somatosensory cortex
SSp-bfd, Primary somatosensory barrelfield cortex
SSs, secondary somatosensory cortex
STP, serial two-photon
Str, striatum
SVZ, subventricular zone
TEa, temporal association area
TF, transcription factor
Th/THAL/Thal, thalamus
TM, tamoxifen
TRE, tetracycline-responsive promoter element
tTA, tetracyclin controlled transactivator
VISp, Primary visual cortex
VM, ventromedial nucleus of the thalamus
VPM, ventral posteromedial nucleus of the thalamus

VZ, ventricular zone
ZI, zona incerta

SUPPLEMENTARY TABLES

Supplementary Table 1 Newly generated mouse driver lines targeting cortical PyNs.
Supplementary Table 2 Comparison of new and existing driver lines.
Supplementary Table 3 Summary of cell distribution experiments acquired by traditional histology.
Supplementary Table 4 Summary of cell distribution datasets acquired by STPT.
Supplementary Table 5 Summary of projection datasets acquired by STPT.
Supplementary Table 6 Full list of SSp-bfd axon targets and values measured from automated detection. Values report number pixels reported per brain structure per dataset on right and left hemisphere.
Supplementary Table 7 Summary of projection datasets acquired by traditional histology.
Supplementary Table 8 List of Videos acquired by STPT

SUPPLEMENTARY VIDEOS

Supplementary Video 1. Whole-brain STP image stack from Cux1-2A-CreER::Ai14 mouse registered to CCFv3.

Supplementary Video 2. Whole-brain STP image stack of anterograde axon projection from right somatosensory cortex from Cux1-2A-CreER;LSL-Flp mouse registered to CCFv3.

Supplementary Video 3. Whole-brain STP image stack from PlxnD1-2A-CreER;Snap25-LSL-EGFP mouse registered to CCFv3.

Supplementary Video 4. Whole-brain STP image stack from PlxnD1-2A-Flp;FSF-tdTomato mouse registered to CCFv3.

Supplementary Video 5. Whole-brain STP image stack of anterograde axon projection from right somatosensory cortex from PlxnD1-2A-CreER;LSL-Flp mouse registered to CCFv3.

Supplementary Video 6. Whole-brain STP image stack from Fezf2-2A-CreER;Ai14 mouse registered to CCFv3.

Supplementary Video 7. Whole-brain STP image stack of anterograde axon projection from right somatosensory cortex from Fezf2-2A-CreER;LSL-Flp mouse registered to CCFv3.

Supplementary Video 8. Whole-brain STP image stack from Adcyap1-2A-CreER;LSL-h2b-GFP mouse registered to CCFv3.

Supplementary Video 9. Whole-brain STP image stack of anterograde axon projection from right somatosensory cortex from Adcyap1-2A-CreER;LSL-Flp mouse registered to CCFv3.

Supplementary Video 10. Whole-brain STP image stack from Tcerg1l-2A-CreER;LSL-h2b-GFP mouse registered to CCFv3.

Supplementary Video 11. Whole-brain STP image stack of anterograde axon projection from right somatosensory cortex from Tcerg1l-2A-CreER mouse registered to CCFv3.

Supplementary Video 12. Whole-brain STP image stack from Sema3E-CreER;Ai14 mouse registered to CCFv3.

Supplementary Video 13. Whole-brain STP image stack from Tbr1-2A-CreER;Ai14 mouse registered to CCFv3.

Supplementary Video 14. Whole-brain STP image stack of anterograde axon projection from right somatosensory cortex from Tbr1-2A-CreER;LSL-Flp mouse registered to CCFv3.

Supplementary Video 15. Whole-brain STP image stack from Tle4-2A-CreER;Snap25-LSL-EGFP mouse registered to CCFv3.
Supplementary Video 16. Whole-brain STP image stack of anterograde axon projection from right somatosensory cortex from Tle4-2A-CreER;LSL-Flp mouse registered to CCFv3.
Supplementary Video 17. Whole-brain STP image stack from Foxp2-IRES-Cre expressing AAV9-CAG-DIO-EGFP by systemic injection registered to CCFv3.
Supplementary Video 18. Whole-brain STP image stack of anterograde axon projection from right somatosensory cortex from Foxp2-Cre mouse registered to CCFv3.

SUPPLEMENTARY NOTES

Instructions for visualizing data on Brain Architecture portal.

High resolution data visualization of STPT cell distributions and axon projections

We have characterized a broad range of glutamatergic cortical pyramidal neuron (PyN) mouse driver knockin lines. Anterograde projection and cell distribution datasets can be visualized on the Mouse Brain architecture website (http://brainarchitecture.org/cell-type/projection and http://brainarchitecture.org/cell-type/density, respectively) as detailed in Supplementary Tables 4, 5. Signal-to-noise level varies from one line to another from line to line but also within an individual brain from brain area to brain area.

Using serial two photon tomography (STPT), a whole-brain fluorescence microscopy approach, we acquired series of 16-bit depth images, providing a very large dynamic range, allowing the imaging parameters to be optimized for stronger or weaker signal.

To visualize the resulting image datasets, the imaging dynamic range must be adjusted, according to the signal level for each mouse driver knockin line, and to optimize signal for a given brain region of interest, when the variability within the dataset is high. Furthermore, due to variability in the imaging parameters (e.g. temperature variability), there may be adjustments to be made even between datasets of a given mouse driver knockin line. We are providing the full dynamic range for the image datasets to allow the user to adjust the signal level as needed. Below we provide a brief manual to assist in adjusting the signal levels on our web portal, brainarchitecture.org.

<u>Changing contrast on the brain architecture web portal</u>

When browsing through high resolution STPT data (using the links provided within the Supplemental Tables 4 and 5), the user may need to adjust contrast.

Click the **Controls** panel 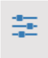 on the upper left-hand corner of the webpage. A pop-out menu will appear.

Drag the upper ‘fluo intensity range’ to an appropriate level, typically 1000-10000, and click **Resample**.
