# Supplementary material for: Genetic dissection of the glutamatergic neuron system in cerebral cortex
Source: Nature. 2021 Oct 6;598(7879):182–7. doi: 10.1038/s41586-021-03955-9 (PMC8494647; doi:10.1038/s41586-021-03955-9)
Supplement: Supplementary file 2 — Reporting Summary [file 41586_2021_3955_MOESM2_ESM.pdf]

nature research

Corresponding author(s): Z. Josh Huang

Last updated by author(s): Jul 30, 2021

Reporting Summary

Nature Research wishes to improve the reproducibility of the work that we publish. This form provides structure for consistency and transparency in reporting. For further information on Nature Research policies, see our Editorial Policies and the Editorial Policy Checklist.

Statistics

For all statistical analyses, confirm that the following items are present in the figure legend, table legend, main text, or Methods section.

n/a	Confirmed	
☐	☒	The exact sample size (*n*) for each experimental group/condition, given as a discrete number and unit of measurement
☐	☒	A statement on whether measurements were taken from distinct samples or whether the same sample was measured repeatedly
☐	☒	The statistical test(s) used AND whether they are one- or two-sided<br>*Only common tests should be described solely by name; describe more complex techniques in the Methods section.*
☒	☐	A description of all covariates tested
☒	☐	A description of any assumptions or corrections, such as tests of normality and adjustment for multiple comparisons
☐	☒	A full description of the statistical parameters including central tendency (e.g. means) or other basic estimates (e.g. regression coefficient) AND variation (e.g. standard deviation) or associated estimates of uncertainty (e.g. confidence intervals)
☐	☒	For null hypothesis testing, the test statistic (e.g. F, t, r) with confidence intervals, effect sizes, degrees of freedom and P value noted<br>*Give P values as exact values whenever suitable.*
☒	☐	For Bayesian analysis, information on the choice of priors and Markov chain Monte Carlo settings
☒	☐	For hierarchical and complex designs, identification of the appropriate level for tests and full reporting of outcomes
☒	☐	Estimates of effect sizes (e.g. Cohen's *d*, Pearson's *r*), indicating how they were calculated

Our web collection on statistics for biologists contains articles on many of the points above.

Software and code

Policy information about availability of computer code

Data collection	1. Serial two photon tomopraphy (STP) datasets were collected using the commercial set-up by TissueVision (Cambridge, MA), followed by Fiji-based stitching, Elastix-based registration, segmentation, quantification and data presentation, as described in Ragan et al 2012 and Mandelbaum et al 2019, doi: 10.1016/j.neuron.2019.02.035, and publicly distributed in Kim et al., 2017, doi: 10.1016/j.cell.2017.09.020).<br>2. For non-STP data, imagesets were acquired (every other section) across the whole brain using a Zeiss Axioimager M2 System equipped with MBF Neurolucida Software (MBF) and x5 objective. Representative fields of view were selected and then confocal image stacks were acquired on a Zeiss LSM 780 or 710 microscope (CSHL St. Giles Advanced Microscopy Center) using objectives x20, x40 and x63.<br>3. Molecular characterization of driver lines with embryonic induction and fixation at P7 was performed on a Nikon Eclipse 90i fluorescence microscope with a x20 objective.<br>4. Electrophysiology data was collected using the commercially available pCLAMP 10.3 software (Molecular devices).
Data analysis	1. Elastix was used within the established pipeline to register brains with an average reference brain, either the Allen Institute's CCFv3 and its associated grayscale brain structure annotations (as in Harris et al 2019) or the Osten reference brain published in Ragan et al 2012 for areal distributions and cortical flatmapping as in Kim et al 2015.<br>2. Code to plot cell distribution in cortical flatmaps is publicly distributed in Kim et al 2017, doi: 10.1016/j.cell.2017.09.020).<br>3. Custom Matlab (Matlab_R2018a) code for both cortical depth distributions and anterograde tracing projection matrix is available upon request.<br>4. Other software includes: MS Excel, GraphPad Prism 7, Fiji/ImageJ Version 2.0.0-rc-68/1.52g; Imaris software (Bitplane) version 9.3 and 9.5; Adobe Photoshop CS6.

For manuscripts utilizing custom algorithms or software that are central to the research but not yet described in published literature, software must be made available to editors and reviewers. We strongly encourage code deposition in a community repository (e.g. GitHub). See the Nature Research guidelines for submitting code & software for further information.

Data

Policy information about availability of data

All manuscripts must include a data availability statement. This statement should provide the following information, where applicable:

- Accession codes, unique identifiers, or web links for publicly available datasets
- A list of figures that have associated raw data
- A description of any restrictions on data availability

Raw and stitched whole-brain STP imaging data is available from the BICCN Brain Image Library (BIL) (http://www.brainimagelibrary.org/download.html) at the Pittsburgh Supercomputing Center, based on the accession codes detailed in Supplementary Tables 1 & 2. Anterograde projection datasets can be visualized on the Mouse Brain architecture website (http://brainarchitecture.org/cell-type/projection) as detailed in Supplementary Tables 1 & 2. All accession codes will be included within Supplementary Tables 1 & 2 prior to publication. All other datasets generated during this study are available from the corresponding author upon request.

Field-specific reporting

Please select the one below that is the best fit for your research. If you are not sure, read the appropriate sections before making your selection.

☒ Life sciences ☐ Behavioural & social sciences ☐ Ecological, evolutionary & environmental sciences

For a reference copy of the document with all sections, see nature.com/documents/nr-reporting-summary-flat.pdf

Life sciences study design

All studies must disclose on these points even when the disclosure is negative.

Sample size	Sample sizes were estimated on the basis of previous studies using similar methods and analyses (Oh et al 2014, doi: 10.1038/nature13186.; Kim et al 2015; Harris et al 2019, doi: 10.1038/s41586-019-1716-z). A full list of data acquired is provided in Tables S1 and S2. Based on consistency of results between litters and between individuals within a given litter, a sample size of 2 litters was considered sufficient for all embryonic characterization.
Data exclusions	All analyzed data was included in the study. Prior to STP analysis, datasets were screened for standard quality control by two independent reviewers according to pre-established criteria.
Replication	-The labeling patterns achieved from the strategies described here reveal a high degree of replicability across animals. A full list of data acquired for cell distribution and virus injections is provided in Supplementary Tables 4 and 5, respectively. These tables detail experiments based on knock in Cre/Flp driver lines bred with appropriate reporters for cell distribution analysis and injected with virus for axon projection mapping.<br>-Oh et al 2014 and Harris et al 2019 demonstrated a high degree of replicability across animals for anterograde virus tracing. Based on this rationale, they confidently and comprehensively sampled with n=1 experiment per source area and driver line.<br>-Here we utilized a minimum n=2-3 for each cell distribution and virus tracing experiment in adult animals, and spanning a minimum of 2 litters for short-pulse embryonic experiments. For Sema3E and Tcerg1l, due to the high replicability mentioned above, we analyzed a single successful injection for each driver line.
Randomization	Not relevant because there was no group allocation.
Blinding	Not relevant to this study because there was no group allocation.

Reporting for specific materials, systems and methods

We require information from authors about some types of materials, experimental systems and methods used in many studies. Here, indicate whether each material, system or method listed is relevant to your study. If you are not sure if a list item applies to your research, read the appropriate section before selecting a response.

Materials & experimental systems

n/a	Involved in the study	
☐	☒	Antibodies
☒	☐	Eukaryotic cell lines
☒	☐	Palaeontology and archaeology
☐	☒	Animals and other organisms
☒	☐	Human research participants
☒	☐	Clinical data
☒	☐	Dual use research of concern

Methods

n/a	Involved in the study	
☒	☐	ChIP-seq
☒	☐	Flow cytometry
☒	☐	MRI-based neuroimaging

Antibodies

Antibodies used

Anti-GFP (1:1000, Aves, GFP-1020); anti-RFP (1:1000, Rockland Pharmaceuticals, 600-401-379); anti-mCherry (1:500, OriGene AB0081-500); anti-mKate2 for Brainbow 3.0 (gift of Dr. Dawen Cai, U Michigan); anti-SATB2 (1:20, Abcam ab51502); anti-CTIP2 (1:100, Abcam 18465); anti-CUX1 (1:100, SantaCruz 13024); anti-LDB2 (1:200, Proteintech 118731-AP); anti-Fog2 (1:500, SantaCruz m-247), anti-LHX2 (1:250, Millipore-Sigma ABE1402) and anti-Tle4 (1:300, Santa Cruz sc-365406).

Validation

All antibodies are commonly used in the field and have been validated in previous publications/by the manufacturer, as detailed here:
-anti-GFP (Aves, GFP-1020): validated by manufacturer by immunohistochemistry (1:500) using transgenic mice expressing GFP
-anti-RFP (Rockland Pharmaceuticals, 600-401-379): validated by the manufacturer by immunoelectrophoresis resulting in a single precipitin arc against anti-Rabbit Serum and purified and partially purified Red Fluorescent Protein (Discosoma). No reaction was observed against Human, Mouse or Rat serum proteins. https://rockland-inc.com/store/Antibodies-to-GFP-and-Antibodies-to-RFP-600-401-379-O4L_24299.aspx
-anti-mCherry (OriGene AB0081-500): https://www.origene.com/catalog/antibodies/primary-antibodies/ab0081-500/mcherry-goat-polyclonal-antibody
-anti-mKate2 for Brainbow 3.0 (gift of Dr. Dawen Cai, U Michigan): validated in Cai et al 2013 (doi: 10.1038/nmeth.2450)
-anti-SATB2 (Abcam ab51502): https://www.abcam.com/satb2-antibody-satba4b10-c-terminal-ab51502.html
-anti-CTIP2 (Abcam 18465): https://www.abcam.com/ctip2-antibody-25b6-chip-grade-ab18465.html
-anti-CUX1 (SantaCruz 13024): https://www.scbt.com/scbt/product/cdp-antibody-m-222
-anti-LDB2 (Proteintech 118731-AP): https://www.ptglab.com/Products/LDB2-Antibody-11873-1-AP.htm#datasheet
-anti-FOG2 (SantaCruz m-247): widely used in the field (e.g. Alfano et al 2014, doi: 10.1038/ncomms6632)
-anti-LHX2 (Millipore-Sigma ABE1402): https://www.emdmillipore.com/US/en/product/Anti-LHX2-Antibody,MM_NF-ABE1402
-anti-Tle4 (SantaCruz sc-365406): https://www.scbt.com/p/tle4-antibody-e-10

Animals and other organisms

Policy information about studies involving animals; ARRIVE guidelines recommended for reporting animal research

Laboratory animals

-Species/strain: Mus musculus, C57Bl/6J or Swiss Webster
Cre/Flp driver knock in and reporter lines used: Lhx2-2A-CreER (JAX stock # 036293), PlexinD1-2A-CreER (JAX stock # 036294), PlexinD1-2A-flpO (JAX stock # 036295), Fezf2-2A-CreER (JAX stock # 036296), Fezf2-2A-flpO (JAX stock # 036297), Tcerg1l-2A-CreER (JAX stock # 034000), Adcyap1-2A-CreER (JAX stock # 033999), Sema3E-CreER (Y. Yoshida; Pecho-Vrieseling et al., 2009, doi: 10.1038/nature08000); Tle4-2A-CreER (JAX stock #), FoxP2-IRES-Cre (R. Palmiter, Rousso et al., 2016, doi: 10.1016/j.celrep.2016.04.069), Tbr1-2A-CreER (JAX stock # 036299), Cux1-2A-CreER (JAX stock # 036300), Tbr2-2A-CreER (JAX stock # 036301), Tbr2-2A-FlpER, Tis21-2A-CreER (JAX stock # 036303), dual-tTA (JAX stock # 036304).
Other Cre/Flp driver knockin and reporter lines used: PV-2A-FlpO (JAX stock # 022730), Ai14 (JAX stock # 007908), Ai65 (JAX stock # 021875), LSL-h2b-GFP (He et al 2016), LSL-Flp (JAX stock # 028584), IS (JAX stock # 028582), Snap25-LSL-EGFP (JAX stock # 021879), RGBbow (JAX stock # 028583), Cux2-Cre (Franco et al., Science 2012, RRID:MMRRC_031778-MU), Cux2-CreERT2 (Franco et al., Science 2012, RRID:MMRRC_032779-MU), Ntsr1-Cre_GN220 (Gerfen et al., Neuron 2013), Rasgrf2-T2A-dgFlpO (JAX stock# 029589), Rbp4-Cre_KL100 (JAX stock # 031125), Sepw1-Cre_NP39 (JAX stock # 037622), Sim1-Cre_KJ18 (JAX stock # 031742), Tlx3-Cre_PL56 (JAX stock # 036547)
New driver and reporter lines have been deposited to the Jackson Laboratory for wide distribution.
-Age: embryonic E11.5, E12.5, E13, E13.5, E14.5, E17; postnatal stages P5, P7, 1-6 months
-Sex: males and females
All details appear in Supplementary Tables 1, 3, 4, 5, 7

Wild animals

The study did not involve wild animals.

Field-collected samples

The study did not involve field-collected samples.

Ethics oversight

All experimental procedures were carried out in accordance with NIH guidelines and approved by the Institutional Animal Care and Use Committees of Cold Spring Harbor Laboratory and Harvard University.

Note that full information on the approval of the study protocol must also be provided in the manuscript.
